# Evaluation of plant contamination in metabarcoding diet analysis of a herbivore

**DOI:** 10.1038/s41598-018-32845-w

**Published:** 2018-10-22

**Authors:** Haruko Ando, Chieko Fujii, Masataka Kawanabe, Yoshimi Ao, Tomomi Inoue, Akio Takenaka

**Affiliations:** 1 Center for Environmental Biology and Ecosystem Studies, National Institute for Environmental Studies, 16-2 Onogawa, Tsukuba, Ibaraki, 305-8506 Japan; 2 Tama Zoological Park, 7-1-1 Hodokubo, Hino-shi, Tokyo, 191-0042 Japan

## Abstract

Fecal DNA metabarcoding is currently used in various fields of ecology to determine animal diets. Contamination of non-food DNA from complex field environments is a considerable challenge to the reliability of this method but has rarely been quantified. We evaluated plant DNA contamination by sequencing the chloroplast *trn*L P6 loop region from food-controlled geese feces. The average percentage of contaminant sequences per sample was 1.86%. According to the results of generalized linear models, the probability of contamination was highest in samples placed in wet soil. The proportion of contaminant sequences was lowest at the earliest sampling point and was slightly higher in samples placed in open conditions. Exclusion of rare OTUs (operational taxonomic units) was effective for obtaining reliable dietary data from the obtained sequences, and a 1% cutoff reduced the percentage of contaminated samples to less than 30%. However, appropriate interpretation of the barcoding results considering inevitable contamination is an important issue to address. We suggest the following procedures for fecal sampling and sequence data treatment to increase the reliability of DNA metabarcoding diet analyses: (i) Collect samples as soon as possible after deposition, (ii) avoid samples from deposits on wet soil, and (iii) exclude rare OTUs from diet composition estimations.

## Introduction

Diet analysis based on fecal DNA metabarcoding has been accepted in the field of ecology as a noninvasive, accurate, time and cost-effective tool to determine animal diets^1–4^. Due to the high resolution of the method, various types of food items that are difficult to identify by other traditional methods have been detected^5,6^. This method enabled recent studies to reveal the detailed mechanisms of niche partitioning in multiple sympatric species^7–9^. The method has also revealed some important aspects of wildlife management: food selection of endangered species and animals that may cause feeding damages^10–13^. In addition, studies to estimate the accuracy of the method based on feeding trials of captive animals have been conducted^14–17^. However, in the various components of metabarcoding diet analysis from sampling to bioinformatics, optimization of the method has not been sufficiently attempted.

One major concern about the reliability of fecal DNA metabarcoding is contamination with non-food DNA^2^. To optimize sampling and data analysis, it is important to quantify contaminant DNA sequences and evaluate the field conditions in which contamination easily occurs. Some contaminant DNA sequences can be detected by comparing them with a candidate list of food items of target animals. For example, bacterial DNA sequences can easily be detected because their sequences are very different from those of actual food^18–20^. In diet analyses of seabirds, which forage in the sea, DNA sequences from terrestrial organisms can be identified as contaminants of their feces, which are collected on land^19,20^. Human DNA can contaminate fecal specimens during sampling and laboratory procedures, but sequences of human DNA can be easily identified and excluded from data analyses^21,22^. Two previous studies have evaluated the effects of field conditions under which samples are collected on the results of molecular-based diet analyses^20,23^. Oehm *et al*.^23^ compared amplification success rates of prey DNA from carrion crow *Corvus corone corone* feces among different sample treatments using a taxon-specific marker. The occurrence ratio of amplifiable DNA other than food was also quantified by Oehm *et al*.^23^, who found that the success rate of amplification of food DNA was decreased in feces deposited on soil and exposed to sunlight or rain for five days. McInnes *et al*.^20^ conducted a metabarcoding diet analysis of the shy albatross *Thalassarche cauta* using a universal primer for major animal lineages. They compared the amplification success rates and sequence proportions of food DNA among various conditions of samples and sampling sites. They found that fecal samples collected from dirt substrates showed lower proportions of food DNA and higher proportions of contaminant DNA than did fecal samples collected from rock substrates. However, all of the above studies targeted the diets of predators.

Most metabarcoding diet studies of herbivores have been conducted with mammals^4,8,11,12,24,25^ and birds^5,10,26,27^. However, contamination of herbivore feces with plant DNA sequences is difficult to discern because there are various sources of plant contamination around samples in the field, most of which can be consumed by the target animals. Thus, few studies have estimated plant DNA contamination from sampling sites. To reliably interpret metabarcoding results while considering plant DNA contamination, it is important to evaluate the possible amounts of contaminant sequences in fecal samples of herbivores. To optimize sampling strategies, it seems essential to identify the conditions that may increase the risk of contamination.

Generally, plant DNA contamination may occur when plant tissue from soil or plants is mixed into fecal samples. This type of contamination can occur with any sample left outside; however, the amount of contamination may be minimized by optimizing the sampling strategy. One approach to control for contamination after it occurs is applied during data analysis. If sequences from contamination are at low frequencies in each sample, the removal of rare OTUs (operational taxonomic units), i.e., OTUs with low sequence proportions, may be effective for removing possible contamination. Several studies have set cutoff values for rare OTUs or detected taxa to exclude sequences that result in sequence errors or contamination^5,8,10,28,29^. However, the effect of rare OTU removal on the removal of contamination has rarely been estimated experimentally. To set appropriate cutoff values for rare OTU removal, interactions between cutoff values and contamination frequencies must be estimated. However, if large amounts of plant tissues are contaminating samples, the estimated diet composition may be greatly affected. Although this type of contamination is difficult to reduce by optimizing sampling and data analysis strategies, it is important to estimate the frequency of such contamination to confirm the reliability of metabarcoding dietary data.

This study quantified plant DNA contamination in a metabarcoding diet analysis of herbivores using food-controlled fecal samples. To identify factors that affect the degree of contamination, we controlled the overhead and ground-surface conditions where the samples were deposited and the amount of time the samples were left at each experimental site. We also proposed appropriate sampling and data analysis strategies to reduce contamination on dietary data.

## Results

After sequence filtering and chimera removal, data from 278 samples (53 control and 225 treatment samples), including 1,155,341 sequence reads, were obtained. The average number of sequence reads per sample was 4,156 ± 4,257 (SD). We defined “non-food OTUs” as OTUs without hits to the fed cabbage *Brassica oleracea* in the BLAST search, and the sequences that were assigned to non-food OTUs were designated “non-food sequences.” Of the sequenced reads, 1,119,946 sequences were assigned to food taxa, and 35,395 were assigned to non-food sequences. OTUs that were not included in the control samples were defined as “contaminant OTUs,” and the sequences that were assigned to contaminant OTUs were designated “contaminant sequences.” The control samples had been stored in a freezer without exposure to the field environment.

**Figure 1:**
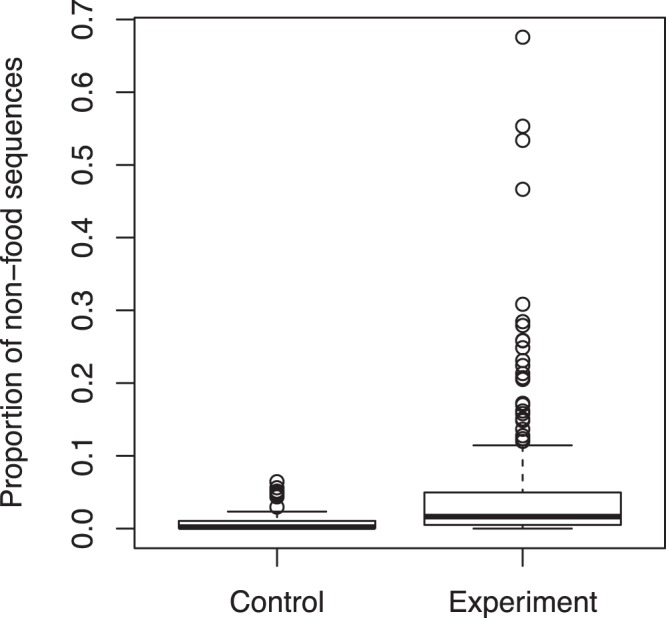
Non-food sequence proportions of the control and treatment samples.

The percentage of non-food sequences ranged from 0.0–6.4% among the control samples, with an average of 1.1 ± 0.2% (SD), and from 0.0–67.6% in the treatment samples, with an average of 5.0 ± 9.1% (SD). The percentage of non-food sequences was significantly larger in the treatment samples than in the control samples (*P* = 2.326E-7, Wilcox test) (Fig. 1). For the treatment samples, the percentage of contaminant sequences, which were sequences not detected in the control samples, ranged from 0.0–46.6%, with an average of 1.9 ± 4.5% (SD). Contaminant sequences were detected in 76% (161 samples) of the 225 treatment samples (Table S1). The sequence percentage of each contaminant OTU per sample ranged from 0.0–44.5%. Among the 110 contaminant OTUs, 81 had sequence percentages less than 1% in all samples.

**Figure 2:**
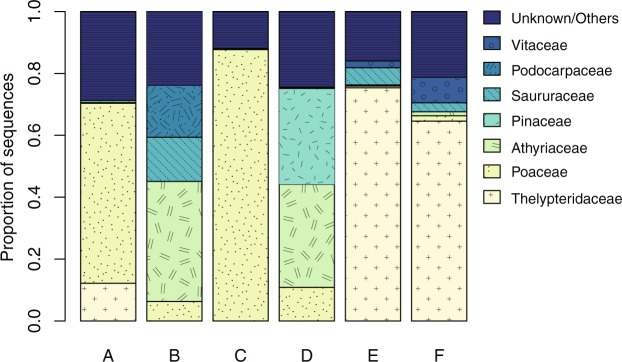
Composition of plant taxa detected from contaminant sequences in samples from six experimental conditions.

The results of the BLAST search detected 52 taxa from contaminant OTUs and 18 taxa from control OTUs at the taxonomic level of family or higher (Table S2). The most dominant taxa in the treatment conditions were Poaceae in A and C, Athyriaceae in B and D, and Thelypteridaceae in E and F (Fig. 2). Poaceae plants covered the samples in conditions A and C and directly contacted the samples in C, whereas Athyriaceae and Thelypteridaceae, which are ferns, neither covered nor contacted any of the samples.

**Figure 3:**
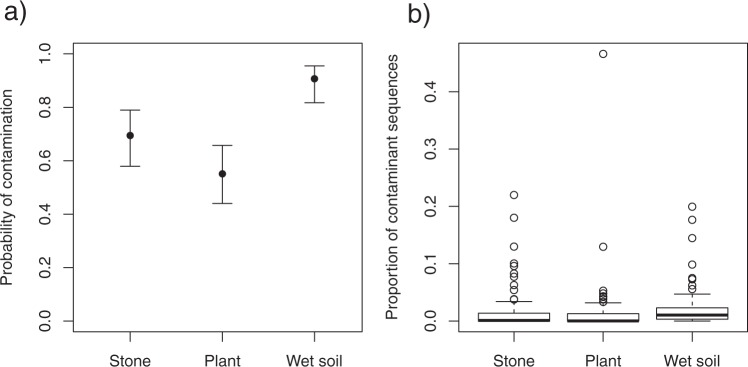
Probabilities of contamination and proportions of contaminant sequences among ground conditions (stone, plant, wet soil). (**a**) Mean predicted values and 95% confidence intervals for the probability of occurrence of contaminated samples predicted by the best-fit generalized linear model. (**b**) Box plot of the proportions of contaminant sequences.

**Figure 4:**
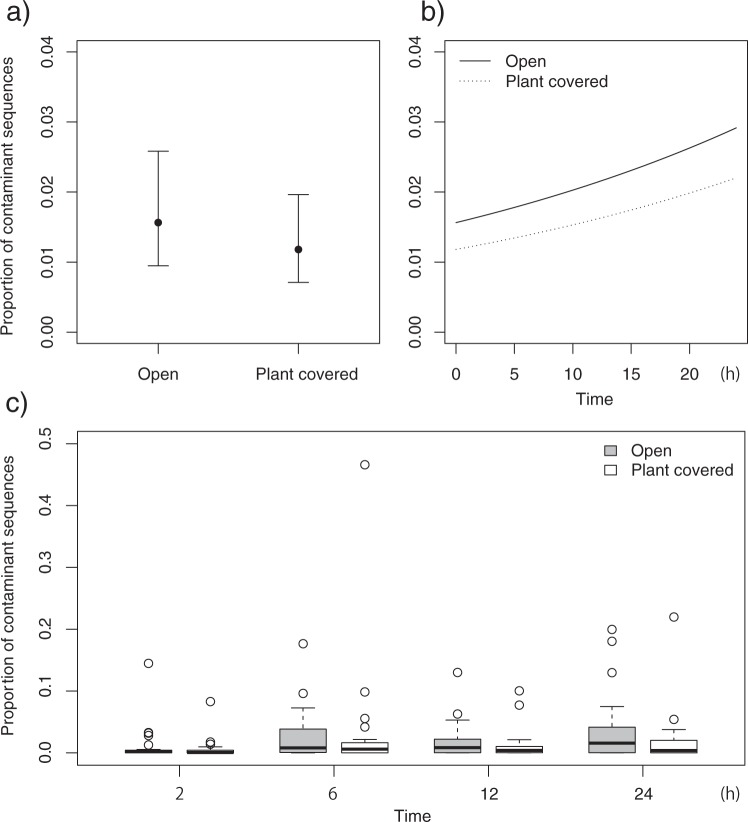
Proportions of contaminant sequences under different overhead conditions (open, plant covered). (**a**) Mean predicted values and 95% confidence intervals predicted by the best-fit generalized linear model. (**b**) Regression curves of the best-fit GLM of contaminant-sequence proportion as a function of time since the samples were placed on the experimental plots. (**c**) Box plot of contaminant-sequence proportion for each overhead condition and time.

To further investigate the factors that affect the degree of contamination, we compared GLMs from presence of contaminated samples and percentage of contaminant sequences as response valuables, and overhead condition, ground condition and amount of time that the sample was left in the plot as explanatory valuables. All of the selected GLMs used to detect the presence of contaminated samples with ΔAIC <2 retained ground condition as an explanatory variable (Tables 1 and S3). The probability of occurrence of contaminated samples was significantly higher for wet soil than for the other ground categories (Figs 3a and S1). The proportion of contaminant sequences was also higher for the wet soil condition than for the other conditions (Fig. 3b). In the analysis of the percentage of contaminant sequences, all of the selected models with ΔAIC <2 included overhead condition as an explanatory variable (Tables 1 and S3). The AIC value was lowest for the model that included overhead condition and time. Although the 95% credible intervals of the estimated values overlapped between the two categories of overhead condition (Fig. 4a), the percentage of contaminant sequences tended to be higher in the open condition, with an average of 2.2 ± 3.9% (SD) under the open condition and of 1.6 ± 5.0% (SD) under the plant-covered condition (Figs 4a,c, S2, S3). However, the two samples with contaminant sequence percentages greater than 20% were both from plant-covered conditions (Fig. S3). These two samples contained contaminant OTUs with sequence percentages of 11.7 and 44.5%; the contaminant OTUs were identified as representing Poaceae taxa that covered the samples in the treatment sites. The regression curve of the best-fit model showed an increase in the percentage of contaminant sequences with time (Fig. 4b), and strong increases in the percentages of contaminant sequences and contaminated samples were observed from two to six hours (Figs 4c, S4, S5).

**Table 1:** Results of the generalized linear models (GLMs) predicting the presence of contaminated samples and the proportions of contaminant sequences.

Response variables	Adopted factors			AIC	ΔAIC
Presence of contaminated samples		Ground		248.47	
		Ground	Time	250.21	1.74
	Overhead	Ground		250.27	1.8
Proportion of contaminant sequences	Overhead		Time	1842.7	
	Overhead			1843.1	0.4

Akaike information criterion (AIC), difference from the smallest AIC value (ΔAIC).

**Figure 5:**
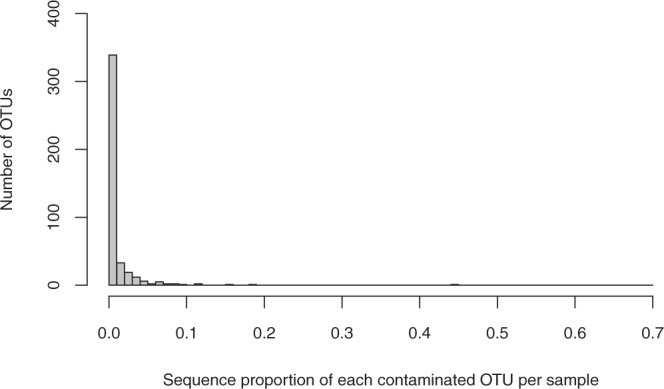
Frequency distribution of the sequence proportions of each contaminant OTU per sample.

**Figure 6:**
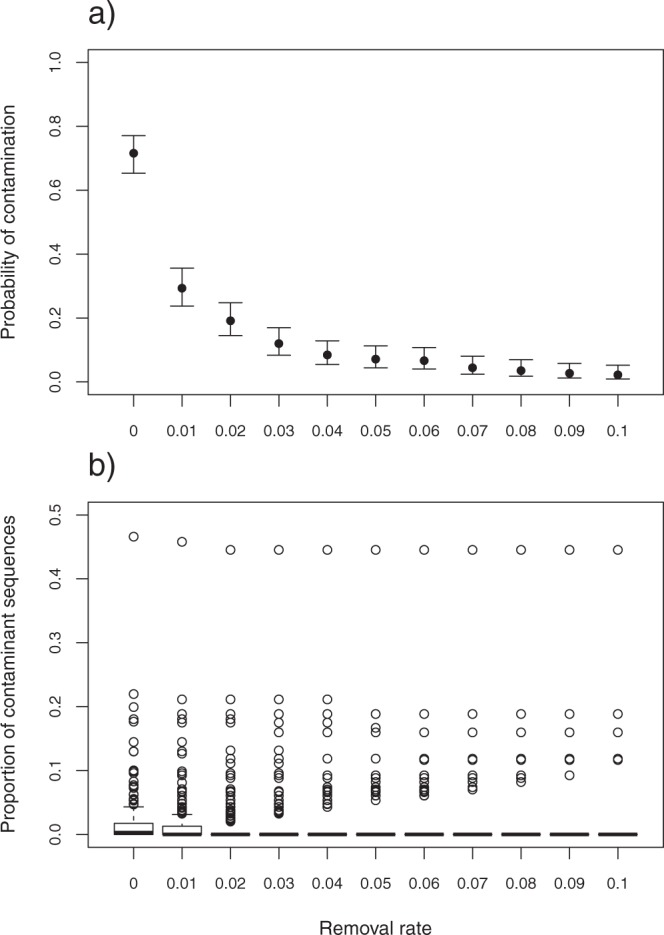
Probabilities of contamination and proportions of contaminant sequences among eleven cutoff values of rare OTU removal. (**a**) Mean predicted values and 95% confidence intervals for the probability of occurrence of contaminated samples predicted by the generalized linear model. (**b**) Box plot of the proportions of contaminant sequences.

Although the contaminant OTUs with sequence percentages below 1% occurred at the highest frequencies, the sequence percentages of five of the contaminant OTUs were greater than 10% (Fig. 5). Across all of the treatment samples, the percentage of contaminated samples ranged from 2.2% (10% cutoff) to 71.6% (0% cutoff). A significant decrease in the probability of contamination occurred between the 0% cutoff and the 1% cutoff (Fig. 6a), and the same tendencies were observed in all of the environmental conditions (Fig. S6). The percentage of contaminant sequences decreased from 1.6% (0% cutoff) to 0.14% (10% cutoff). The pattern of decrease in the contaminant sequence percentage differed among the environmental conditions (Fig. S6). Except for five samples that were highly contaminated by specific OTUs, contaminant sequences were completely excluded at the 10% cutoff (Figs 6b and S6).

## Discussion

This study quantified the contamination of plant DNA in a metabarcoding diet analysis of a herbivore. Such contamination has been difficult to estimate for fecal samples of wild animals. Although contaminant sequences occurred at low frequencies in most cases, contaminant sequences were detected in most of the fecal samples in this study, and the degree of contamination varied with time and environmental condition.

The probability of occurrence of contaminated samples was highest under the wet soil condition. Similarly, McInnes *et al*.^20^ found lower proportions of food-DNA sequences and higher proportions of contaminant (e.g., unicellular) DNA sequences in samples collected from a dirt surface than in those collected from a rocky surface. In the present study, which targeted vascular plants, small amounts of plant tissue or spores might have been mixed into the samples via water. Sequences of ferns were frequently detected in the samples placed on wet soil. We confirmed that ferns grew around the sampling sites but did not cover any of the samples in this experiment. This indicates that spores of ferns contaminated the samples via water. Soil containing the remains of plant tissue from the surrounding area might also have been collected together with the fecal samples. Hence, plant DNA contamination of fecal samples may easily occur on wet soil, regardless of the time since fecal deposition. In the wet soil condition, most of the samples were contaminated, and nearly 80% of the samples showed more than 0.5% of contaminant sequences (Fig. 3b). This finding suggests that diet composition estimates may be affected, especially when rare OTUs are included in the data analyses.

The samples in the open condition showed higher proportions of contaminant sequences than did the samples in the plant-covered condition, which could suggest contamination from plant tissues, pollen or spores delivered by wind. As in the wet soil condition, in the open condition, fern sequences were frequently detected in the samples, regardless of the ground conditions. This indicates that fern spores can readily contaminate fecal samples via water or wind and can be misinterpreted as food. Furthermore, the two samples in the plant-covered condition that had high percentages of contaminant sequences (>20%) were contaminated by single OTUs of Poaceae taxa. This result indicates that large amounts of tissue from plants that covered the samples contaminated the fecal samples. In this case, the estimated sequence compositions are very different from the actual diet composition due to plant contamination. Such highly contaminated samples may lead to error in the estimation of dietary similarity or food selectivity among individuals (samples). If the diet compositions of some samples are very different from those of others collected in a similar environment, the possibility of plant contamination should be considered.

The proportion of contaminant sequences increased with exposure time. The effects of sample freshness on the results of fecal DNA analyses have been discussed in several studies. For example, the success rate of genotyping target species has been found to decrease over time (days)^30–32^. Regarding the detection of food DNA in feces, McInnes *et al*.^20^ and Oehm *et al*.^23^ found that the amplification success rate was lower in older samples than in newer ones in a field environment. In agreement with the above studies, this study demonstrates the importance of collecting fresh samples not only to increase the success rate of target DNA detection but also to reduce DNA contamination. In addition, this study examined the results obtained within a short period of time since fecal deposition (within 24 hours); such a time period was not considered in any of the above-mentioned studies except McInnes *et al*.^20^. In McInnes *et al*.^20^, “fresh” samples, which were collected after direct observation of defecation, showed higher amplification success rates of food DNA and higher proportions of food DNA sequences than did older samples. The contamination estimated in this study was lowest when the samples were collected within two hours after being placed outside, which we recommend as the time interval after defecation within which samples should be collected. Although the estimated barcoding regions were different between McInnes *et al*.^20^ and the present study, the results of both studies highlight the importance of collecting samples soon after defecation to increase the reliability of fecal metabarcoding results.

Plant DNA contamination can be reduced by taking precautions during sampling and considering the above-mentioned factors that affect the degree of contamination. However, the complete exclusion of contamination is difficult. In this study, most of the contaminant OTUs showed sequence percentages of less than 1% per sample (Fig. 5). Thus, the exclusion of rare OTUs can be used to effectively obtain reliable dietary data from obtained sequences. Although the effect of rare OTU removal differed among the environmental conditions, some general cutoff values can be suggested. For example, the percentages of contaminated samples were reduced to approximately 30% at the 1% cutoff and to approximately 5% at the 7% cutoff (Fig. 6a). At the 10% cutoff, contaminant sequences were excluded from all but five of the samples. To exclude contaminant sequences from more than 80% of samples, a 3% cutoff is sufficient (Figs 6 and S6).

There have been several arguments regarding the quantification of diet using the number of sequence reads obtained from next-generation sequencers; the number of sequence reads does not precisely reflect the amount of food due to various biological and technical biases^2,15,33,34^. Thus, some studies have analyzed diet composition using presence/absence data of individual food items in each sample^10,18,22,35^. However, with this approach, contaminant OTUs with low sequence proportions may be misidentified as food. Contaminant OTUs that are present in many samples can be erroneously inferred to be main food items due to their high frequencies of occurrence. Some studies have indicated positive correlations between sequence proportion and mass proportion of the actual diet^25^; thus, recent studies have tended to use sequence proportions as semi-quantitative indicators of diet composition^8,19,25^. This method seems to be appropriate for avoiding the misidentification of contaminant OTUs as food and to exclude possible contamination by rare OTU removal. However, it should be noted that some information on actual food items may be lost by high cutoff values (e.g., more than 1%).

Some samples showed high proportions of contaminant sequences corresponding to a specific OTU, which cannot be excluded by rare OTU removal (Figs 5, 6 and S6). This type of contamination may accidentally occur and is difficult to discriminate from non-contaminant DNA. In sample-based analyses, the possibility of contamination should be considered when some of the samples show dietary compositions different from those of other samples collected from similar environments. When data from many samples are pooled for diet comparisons among populations (or species), the effects of such abnormal samples may be weak. After rare OTU removal, it may be useful to calculate both the sequence proportion and frequency of occurrence of each detected plant among all samples to distinguish among general food, rare food and contaminants.

This study quantified plant DNA contamination in a metabarcoding diet analysis of a herbivore. We also estimated the factors that affect the degree of DNA contamination in fecal samples. Based on our results, we suggest the following procedures for sampling and data analyses to minimize plant DNA contamination and its effects on the results of diet analyses. (1) Collect fecal samples as soon as possible after defecation. (2) Avoid collecting samples from feces deposited on wet soil. If samples are collected in such conditions, special care is required to not include plant fragments in the samples. (3) Remove rare OTUs from the diet composition data analyses. The cutoff values suggested in this study can be the basis for the exclusion of possible contamination. The results of this study can contribute to increasing the reliability of metabarcoding diet analyses, which are becoming increasingly popular in various ecological studies as noninvasive and accurate methods to detect animal diets.

## Methods

### Ethical considerations

Feeding and fecal sampling were carried out in accordance with the guidelines of the National Institute for Environmental Studies Animal Experiment Committee and Science Council in Japan. Since the cabbage fed to the geese was one of their main daily foods before the study, the feeding protocol did not impose any stress upon them. We did not conduct forced feeding and or any other special operations during the feeding or fecal sampling. The protocol of the study was approved by National Institute for Environmental Studies and Tama Zoological Park.

### Feeding protocol and fecal sampling

We fed cabbage *Brassica oleracea* var*. sabellica* to four individuals of a herbivore waterfowl, the lesser white-fronted goose *Anser erythropus*, housed at Tama Zoological Park, Tokyo, Japan. The geese were three months old; two were male, and the other two were female. The geese were housed in a single cage separated from other bird species. Before the feeding procedure, the geese had been fed Japanese mustard spinach *Brassica rapa* var*. perviridis*, cabbage and assorted chicken feed. Since the maximum retention time of the *Anser* goose species has been estimated to be approximately eight hours^36,37^, we changed the goose food to cabbage one day before sample collection. Sampling was conducted on 28 and 29 August 2016. After feeding, the cage was inspected every two hours, and newly defecated feces were collected. To correct for the size variation of the feces, we mixed the collected feces together and placed 2 g samples of feces into individual plastic bags. In total, we used 300 fecal samples in the experiment. The samples were stored at −30 °C before being placed under field conditions.

### Sample exposure to field conditions

**Figure 7:**
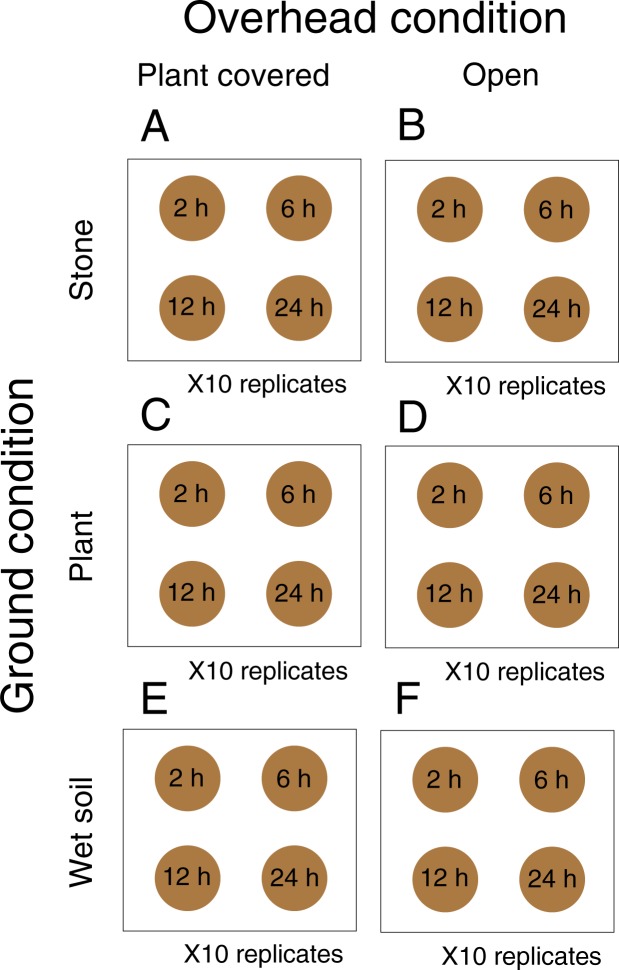
Sampling design of this study. We combined two overhead conditions (open/plant covered) and three ground conditions (stone/plant/wet soil). The gray circles represent fecal samples exposed to each environmental condition, and exposure time is presented within the circles.

The following experiment was conducted at the National Institute for Environmental Studies, Ibaraki, Japan. In consideration of DNA contamination from the ground and from above, we defined six combinations of environmental conditions, A-F (Figs 7 and S7), to which samples would be exposed. We categorized the ground-surface conditions to which samples were directly exposed as “Stone”, “Plant” and “Wet soil”. The samples subjected to C and D conditions were placed on leaves of Poaceae and Asteraceae plants, respectively. The overhead conditions were categorized as “Open” and “Plant covered”, in which no plant material was present above the samples and the samples were situated below plants of a herbaceous community, respectively. The samples subjected to A and C conditions were covered with Poaceae plants, and those under E conditions were mainly covered with Vitaceae, Saururaceae, Asteraceae, and Poaceae plants. For each condition, we set ten experimental plots. We confirmed that no *Brassica* species related to cabbage existed in or around any of the plots. We placed four fecal samples in each plot and collected one sample after two, six, 12 and 24 hours. The remaining 60 samples were stored at −30 °C as control samples. The samples that were collected after exposure to each environmental condition were stored at −30 °C before DNA extraction.

### Amplicon library preparation and Ion PGM sequencing

DNA extraction was performed using the DNeasy Plant Mini Kit (QIAGEN, Venlo, the Netherlands). We used the universal primer pair *g* and *h* designed by Taberlet *et al*.^38^ to amplify the DNA region of the chloroplast *trn*L P6 loop. Due to its high conservativeness, moderate mutation rate of amplicon sequences and short fragment sizes (usually below 150 bp), the *g*-*h* primer pair is widely used in metabarcoding diet analyses of herbivores^2,39^ as universal primer that targets vascular plants. To track the resulting sequences from each sample, the forward primer was tagged with a multiplex identifier (MID)^40^. The detailed procedure of amplicon library preparation is described in Ando *et al*.^5,10^. We performed one run of amplicon sequencing using an Ion Torrent Personal Genome Machine (PGM) system with the Ion PGM^TM^ Hi-Q Sequencing Kit and the Ion 318^TM^ Chip (Thermo Fisher Scientific) following the manufacturer’s instructions.

### Discrimination of contaminant sequences

The following analyses were performed using Claident software^41^, which is a software package for sequence clustering and taxonomic identification in metagenomics. The sequences in each sample were separated based on the MID tags. During the filtering process, sequences that met more than one of the following conditions were excluded from further analysis: (1) sequence length <55 bp, (2) sequence length >200 bp (3) mean quality value <20, and (4) minimum quality value of the MID tags <27. Low-quality 3′ tails were also trimmed until three continuous sequences were obtained with a minimum quality value of 20. Removal of possible chimera sequences was performed using the default settings. After chimera removal, the samples that had fewer than 100 reads were excluded from further analysis. The sequences that remained after filtering and chimera removal were clustered, with those clustering with ≥97% similarity defined as one OTU. For each OTU, NCBI BLAST search was performed based on the Megablast algorithm. Taxa corresponding to each OTU were identified to family or higher taxonomic level based on 95% similarity in the BLAST search. Since we had not developed a local reference database of plants living around the sampling sites, we set the lowest taxonomic level of OTU identification to family to avoid misidentification of genera or species following Nakahara *et al*.^33^. We regarded those OTU sequences with hits with *Brassica oleracea* with at least 95% similarity as “food OTUs”. Other OTUs were defined as “non-food OTUs”, and the sequences that were assigned to each non-food OTU were defined as “non-food sequences.”

We calculated the proportion of non-food sequences per sample and compared the proportions between the control and experimental samples. Non-food sequences were also found in the control samples, which may represent DNA sequences from the food the geese ate more than two days before the feeding procedure or contamination during the laboratory procedures. Since this study focused on DNA contamination from the environment where the fecal samples were placed, the subsequent analyses were performed using only those OTUs that were not found in the control samples. We defined such OTUs as “contaminant OTUs,” and the sequences that were assigned to each contaminant OTU were defined as “contaminant sequences.”

### Estimation of factors that affect the degree of contamination

The following analyses were performed using R ver. 3.3.2^42^. To estimate the factors that affected plant DNA contamination of the fecal samples, we performed two different generalized linear model (GLM) analyses by using the stats and MASS R packages^43^. First, we estimated the factors that were correlated with the presence of contaminant sequences in the samples (contaminated samples) using GLMs with a binomial distribution. The response variable of the model was the presence of contaminated samples including more than one contaminant sequence. The candidate explanatory variables were overhead condition, ground condition and amount of time that the sample was left in the plot. Second, we used binomial GLMs to estimate the factors that affect the proportion of contaminant sequences. The response variable of the model was the proportion of contaminant sequences per sample. The candidate explanatory variables were the same as in the first model. We used the total available sequence reads of the samples as an offset term. Overdispersion was detected in the second analysis, and we corrected the standard errors of the estimates by multiplying them by the square root of the dispersion parameter. Appropriate models were selected based on the Akaike information criterion (AIC). Models with ΔAIC values (difference from the smallest AIC score) of <2 were considered for discussion^44^. We plotted the estimated values of the best-fit models.

### Effect of rare OTU removal on reduction of contaminant sequences

For each sample, the sequence proportion of each contaminant OTU was calculated. To estimate the effect of rare OTU removal on the reduction of contaminant sequences, we set eleven cutoff values for rare OTUs ranging from 0–10% of the sequence proportion per sample. After the rare OTUs under each cutoff value were removed from each sample, we calculated the proportion of contaminated samples and the proportion of contaminant sequences for each environmental condition (A-F). Under each cutoff value, the probability of contamination was estimated by GLM. The response variable of the model was the presence of contaminated samples including more than one contaminant sequence, and the explanatory variable was the cutoff value for rare OTU removal.

## Electronic supplementary material

Supporting information

Table S1

Table S2

Table S3

## Data Availability

FASTA files of each OTU and FASTQ files of each sample separated by MID tags have been archived in Dryad repository (10.5061/dryad.q62rb2p).
