# Supplementary material for: Evaluation of plant contamination in metabarcoding diet analysis of a herbivore
Source: Sci Rep. 2018 Oct 22;8:15563. doi: 10.1038/s41598-018-32845-w (PMC6197254; doi:10.1038/s41598-018-32845-w)
Supplement: Supplementary file 1 — Supporting information [file 41598_2018_32845_MOESM1_ESM.docx]

**Supporting information**

Evaluation of plant contamination in metabarcoding diet analysis of a herbivore

Haruko Ando, Chieko Fujii, Masataka Kawanabe, Yoshimi Ao,

Tomomi Inoue, Akio Takenaka

Fig. S1. Proportions of contaminant samples among ground conditions.

Fig. S2. Proportions of contaminant samples between overhead conditions.

Fig. S3. Proportions of contaminant sequences between overhead conditions.

Fig. S4. Proportions of contaminant samples among time before sampling.

Fig. S5. Proportions of contaminant sequences among time before sampling.

Fig. S6. Plots representing the effects of eleven cutoff values for rare OTU removal on the average proportion of contaminant sequences and contaminated samples for six environmental conditions.

Fig. S7. Schematics of sampling sites.
